# Dual function of *GbNAC2* in flavonoid metabolism and hormonal pathways enhances salt tolerance in *Ginkgo biloba*

**DOI:** 10.48130/forres-0025-0027

**Published:** 2025-11-20

**Authors:** Jinkai Lu, Han Tang, Wei Li, Yanbin Jiang, Helin Zou, Zhili Wang, Weixing Li, Qingjie Wang, Li Wang

**Affiliations:** 1 College of Horticulture and Landscape Architecture, Yangzhou University, Yangzhou 225009, China; 2 Centre for Soybean Research of the State Key Laboratory of Agrobiotechnology and School of Life Sciences, The Chinese University of Hong Kong, Hong Kong Special Administrative Region, China; 3 Shenzhen Research Institute, The Chinese University of Hong Kong, Shenzhen 518057, China

**Keywords:** Ginkgo biloba, Salt stress, NAC transcription factor, Flavonoid, ABA

## Abstract

NAC transcription factors are central regulators of plant salt tolerance, yet their specific roles in ginkgo salt response remain unclear. Here, *GbNAC2* was identified as a salinity-induced transcriptional activator in ginkgo, orchestrating two key adaptive responses. *GbNAC2* overexpression significantly improved salt tolerance in transgenic plants, accompanied by over 60% increase in root length, and more than 20% increase in flavonoid content compared to wild type (WT). Transcriptome analysis of *GbNAC2*-overexpressing ginkgo calli revealed that genes related to auxin biosynthesis, and those involved in the flavonoid synthesis pathway, were significantly upregulated in transgenic calli. Mechanistically, GbNAC2 directly binds the *GbAREB3* promoter to enhance ABA signaling, and exogenous ABA treatment further enhances salt resilience. The present findings unveil a unique crosstalk mediated by GbNAC2 between flavonoid-antioxidant systems and auxin-ABA hormonal networks, effectively resolving the growth-defense trade-off under salinity in ginkgo.

## Introduction

Soil salinity is a major abiotic stress that severely disrupts plant growth by impairing root development, inducing oxidative damage, and destabilizing hormonal homeostasis^[1]^. To mitigate salt toxicity, plants deploy a variety of strategies, including architectural remodeling of roots, accumulation of osmoregulatory metabolites, and activation of antioxidant systems^[2]^. As the primary organ sensing salinity, the root perceives salt stress and responds by promoting downward growth to access deeper soil layers, thereby enhancing water and nutrient acquisition, while simultaneously limiting salt influx^[3]^. Flavonoids function as non-enzymatic antioxidants that are pivotal for scavenging reactive oxygen species (ROS), and maintaining redox equilibrium under stress^[4,5]^.

Plant growth and stress responses are tightly regulated by phytohormones, which coordinate physiological adaptations to environmental cues. Under salt stress, hormonal networks, especially the interplay between auxin-mediated growth regulation and abscisic acid (ABA)-driven stress responses, are crucial for maintaining cellular homeostasis^[6]^. Auxin, a key regulator of plant development, mediates root architectural adjustments essential for nutrient acquisition in saline soils^[7]^. Meanwhile, ABA acts as a central hub for stress tolerance, orchestrating stomatal closure to curtail water loss and simultaneously driving root elongation to enhance water acquisition^[8,9]^. The crosstalk between antioxidant pathways and hormonal signaling is critical for balancing the inherent trade-off between growth and stress defense in plants facing salinity.

Plants coordinate biochemical reactions and metabolic pathways through a complex network of key genes. NAC transcription factors (TFs) function as master regulators that integrate these adaptive responses, enhancing stress resilience by directly activating antioxidant biosynthesis genes and bridging with hormonal pathways^[10]^. For example, NAC TFs enhance ABA signaling to improve stomatal closure and ROS detoxification, while simultaneously facilitating auxin-driven root plasticity in species such as *Vitis vinifera* and poplar^[11]^. In addition, the *Arabidopsis* NAC gene *SMB* regulates *AUX1* expression, thereby activating auxin signaling and root halophytic responses^[12]^. However, the mechanisms by which NAC proteins coordinate flavonoid-antioxidant systems with auxin-ABA crosstalk to balance salt adaptation in economically important tree species remain unclear.

*Ginkgo biloba*, a medicinal tree highly valued for its flavonoid content, exhibits severe growth suppression under saline conditions. Although NAC TFs such as *GbNAC2* have been previously identified as salt-responsive genes in ginkgo^[13]^, their functional mechanisms in integrating antioxidant and hormonal pathways remain unexplored. In the present study, *GbNAC2* was identified as a critical salinity-tolerance gene, revealing its ability to enhance salt resistance through multiple synergistic strategies. Specifically, *GbNAC2* promotes flavonoid accumulation to strengthen antioxidant defenses, modulates auxin signaling to optimize root architecture for stress adaptation, and activates ABA-mediated pathways to amplify salt resilience. These findings provide critical insights into the molecular mechanisms underlying salt tolerance in ginkgo, serving as a valuable genetic resource for the development of salt-tolerant ginkgo through biotechnological breeding.

## Materials and methods

### Plant materials and treatments

Surface-sterilization of Ginkgo seeds (Fozhi cultivar) was performed using 15% sodium hypochlorite for 15 min, followed by three rinses with sterile water. Subsequently, the seeds were placed in an incubator set at 25 °C for a 10-d germination period. Seeds with uniform germination were chosen and transplanted into pots containing nutrient-dense soil. The seedlings were cultivated in a growth cabinet at 25 °C, under a photoperiod of 16 h of light and 8 h of darkness. Ginkgo seedlings cultivated for 2 months, with good growth status and similar plant height and stem diameter, were used for various experiments. The sampling standard for leaves was the middle part of each plant.

For salt treatment, seedlings were treated with 200 mM NaCl as described in previous research^[13]^. For ABA treatment, leaves were sprayed with a 70 μM ABA solution. For combined NaCl and ABA treatment, seedlings were first sprayed with 20 μM ABA, followed by 400 mM NaCl treatment the next day. After 3 d of NaCl treatment, seedlings' phenotypes were observed, and leaf samples were collected for further analysis.

### Phylogenetic analysis

Homologous protein sequences of the GbNAC2 and GbAREB3 from other species were obtained from the National Center for Biotechnology Information (NCBI) database. The protein sequences were aligned using ClustalW, and a neighbor-joining (NJ) phylogenetic tree was constructed using MEGA software^[14]^. The corresponding accession numbers (IDs) of genes are provided in Supplementary Table S1.

### Expression analysis

Total RNA was extracted from various ginkgo tissues (root, stem, leaf, embryo, ovule, and stamen), calli, and transgenic plant leaves utilizing the RNA Extraction Kit (Nanjing, Vazyme). Samples were pulverized into a fine powder in liquid nitrogen, and 0.1 g of this powder was subjected to RNA extraction. The RNA was validated for concentration using NanoDrop 2000 (Thermo Scientific, Waltham, MA, USA). Quantitative real-time PCR (qRT-PCR) analysis was conducted with SYBR qPCR Master Mix (Nanjing, Vazyme), with *G. biloba* GAPDH serving as the reference gene for normalizing relative expression levels. Relative gene expression was computed using the 2^−ΔΔCᴛ^ method^[15]^. The primer sequences used are provided in Supplementary Table S2.

### Subcellular localization

The coding sequence (CDS) of *GbNAC2* was inserted into the pACT2 vector to generate a GbNAC2-green fluorescent protein (GFP) fusion construct (35S::GbNAC2-GFP). The plasmid was transformed into competent cells of the *Agrobacterium tumefaciens* (*A. tumefaciens*) strain GV3101. Subsequently, 1-month-old tobacco plants with healthy growth were selected for transient transformation via infiltration with *Agrobacterium* harboring 35S::GbNAC2-GFP, following the method described by Liu et al.^[16]^. Leaf samples were collected after 48–72 h of dark incubation, and GbNAC2 localization was determined by observing GFP fluorescence using a laser confocal microscope. The primer sequences used are provided in Supplementary Table S2.

### Heterologous overexpression in *Arabidopsis* and poplar

To obtain stable transgenic *Arabidopsis* plants, 1-month-old wild-type (WT) *Arabidopsis* was transformed using the floral dip method as previously described^[17]^. The *Arabidopsis* transformation was conducted using the GV3101 strain of *A. tumefaciens*. For the transformation of '84K' poplar, an *Agrobacterium*-mediated leaf transformation method was used as described by Wen et al.^[18]^. The selected plants were then cultivated for approximately 4 weeks, after which genomic DNA and RNA were extracted for verification of successful transformation.

### Transient overexpression of *GbNAC2* in ginkgo calli

To analyze the downstream genes regulated by *GbNAC2*, the CDS of *GbNAC2* was constructed into the *Bam*HI restriction site of the pRI101-AN vector, yielding the overexpression vector *GbNAC2*-pRI. *Agrobacterium*-mediated transformation was used to introduce the recombinant plasmid into ginkgo calli, following the procedures outlined in a previous study^[19]^. The primer sequences used are provided in Supplementary Table S2.

### Determination of physiological indexes

The detection of flavonoids was performed according to the previous method by Xu et al.^[13]^ using a flavonoid detection kit (Cat#BC1330; Suzhou Conmin Biotechnology Co., Ltd.). Samples were dried to constant weight and extracted using the ultrasonic extraction method (300 Hz, 60 °C, 30 min). After extraction, the mixture was centrifuged at 12,000 rpm at 25 °C for 10 min, and the absorbance of the resulting supernatant was determined at 470 nm via spectrophotometry.

The determination of the contents of hydrogen peroxide (H_2_O_2_) and malondialdehyde (MDA), as well as the determination of the activities of catalase (CAT) and superoxide dismutase (SOD), were all performed according to previous methods^[20]^. Additionally, the ultraviolet spectrophotometry was used in accordance with the manufacturer's instructions (Suzhou Conmin Biotechnology Co., Ltd.). Specifically, CAT (Cat#BC0200), SOD (Cat#BC01700), H_2_O_2_ (Cat#BC3590), and MDA (Cat#BC0020) content assay kits were used respectively. CAT activity was determined at 240 nm, with results expressed as U·g^−1^; SOD activity was assayed at 560 nm, also quantified as U·g^−1^; H_2_O_2_ content was measured at 415 nm, with the unit of μmol·g^−1^; MDA concentration was determined at dual wavelengths of 532 and 600 nm, and expressed as nmol·g^−1^.

### Germination experiment of *Arabidopsis* seeds

Seeds of WT *Arabidopsis* and *GbNAC2* transgenic *Arabidopsis* were surface-sterilized with 15% sodium hypochlorite, and 70% ethanol, respectively. Subsequently, the seeds were evenly sown on 1/2 MS solid medium, 1/2 MS solid medium supplemented with 100 mM NaCl, and 1/2 MS solid medium supplemented with 200 mM NaCl. After stratification at 4 °C for 3 d, the seeds were transferred to a growth chamber at 26 °C. Then, the germination rate was assessed after 7 d.

### Transcriptome sequencing and DEGs analysis

RNA sequencing was performed using ginkgo callus samples with empty vector (WT), and *GbNAC2*-overexpression (*GbNAC2*-OE), with three biological replicates set for each treatment. After the samples were ground into fine powder in liquid nitrogen, total RNA was extracted using the RNAprep Pure Plus Kit (TIANGEN, Beijing, China) according to the operating protocol provided by the manufacturer. RNA integrity was evaluated using the RNA Nano 6000 Assay Kit matched with the Bioanalyzer 2100 System (Agilent Technologies, CA, USA). A total of six libraries were constructed in the experiment, and subsequent sequencing was completed using the Illumina NovaSeq sequencing platform at Novogene (Beijing, China).

Quality control was performed on the raw data to obtain clean reads through the following specific steps: removing adapter sequences from the reads, discarding reads with more than 10% unknown bases (N), and filtering out reads with a sequencing quality score lower than Q20. Filtered clean reads were aligned to the ginkgo reference genome^[21]^. Gene expression levels were quantified as fragments per kilobase of transcript per million mapped reads (FPKM). Differentially expressed genes (DEGs) between the WT and *GbNAC2*-OE groups were identified using the DESeq2 package in R (version 1.16.1), based on *p*-value < 0.05, and fold change ≥ 2. Functional enrichment analysis of the DEGs was conducted according to the KEGG database (www.genome.jp/kegg)^[22]^.

### Transcriptional activation analysis of GbNAC2 protein

The *GbNAC2* CDS was constructed into the *Bam*HI restriction sites of the pGBKT7 (BD) vector following the method described by Hou et al.^[23]^. The BD-GbNAC2 plasmid was co-transformed with the empty vector pGADT7 (AD) into the Y2H Gold yeast strain. Following transformation, the yeast cells were spread onto SD/-Leu/-Trp selective medium (DDO), and incubated at 30 °C for 2–4 d. Subsequently, the yeast strains were transferred into the SD/-Leu/-Trp/-His/-Ade/5-bromo-4-chloro-3-indolyl-*α*-d-galactopyranoside (X-*α*-Gal) selective medium (QDO). The plates were incubated again at 30 °C for 2–4 d, during which the development of the colonies and any color changes were monitored. The appearance of blue yeast strains indicated the presence of transcriptional activation activity. The primer sequences used are provided in Supplementary Table S2.

### Yeast one-hybrid (Y1H) assay

Previous studies have described the methods for performing Y1H^[24]^. The promoter sequence (2000 bp) of *GbAREB3* and the *GbNAC2* CDS were respectively inserted into the pLacZi-2*μ* and pb42AD vectors. The constructs were co-transformed into competent yeast cells (EGY48), which were then plated on selection medium (SD/-Ura/-Trp), and incubated at 28 °C for ~3 d. Yeast monoclonal colonies from the selection media were transferred to a color development medium containing 5-bromo-4-chloro-3-indolyl-*β*-d-galactopyranoside (X-*β*-gal), and Buffer (BU) salt and incubated in a 28 °C incubator for ~3 d. If the target protein binds to DNA, the colonies will exhibit a blue color. The primer sequences used are provided in Supplementary Table S2.

### Statistical analysis

All experiments were performed with at least three biological replicates. Asterisks indicate significant differences by two-sided Student's *t*-test (* *p* < 0.05, ** *p* < 0.01). Different letters indicate significant differences by one-way analysis of variance (ANOVA, *p* < 0.05). Error bars represent the mean ± standard deviation (SD).

## Results

### *GbNAC2* is a putative salt-responsive gene in ginkgo

**Figure 1:**
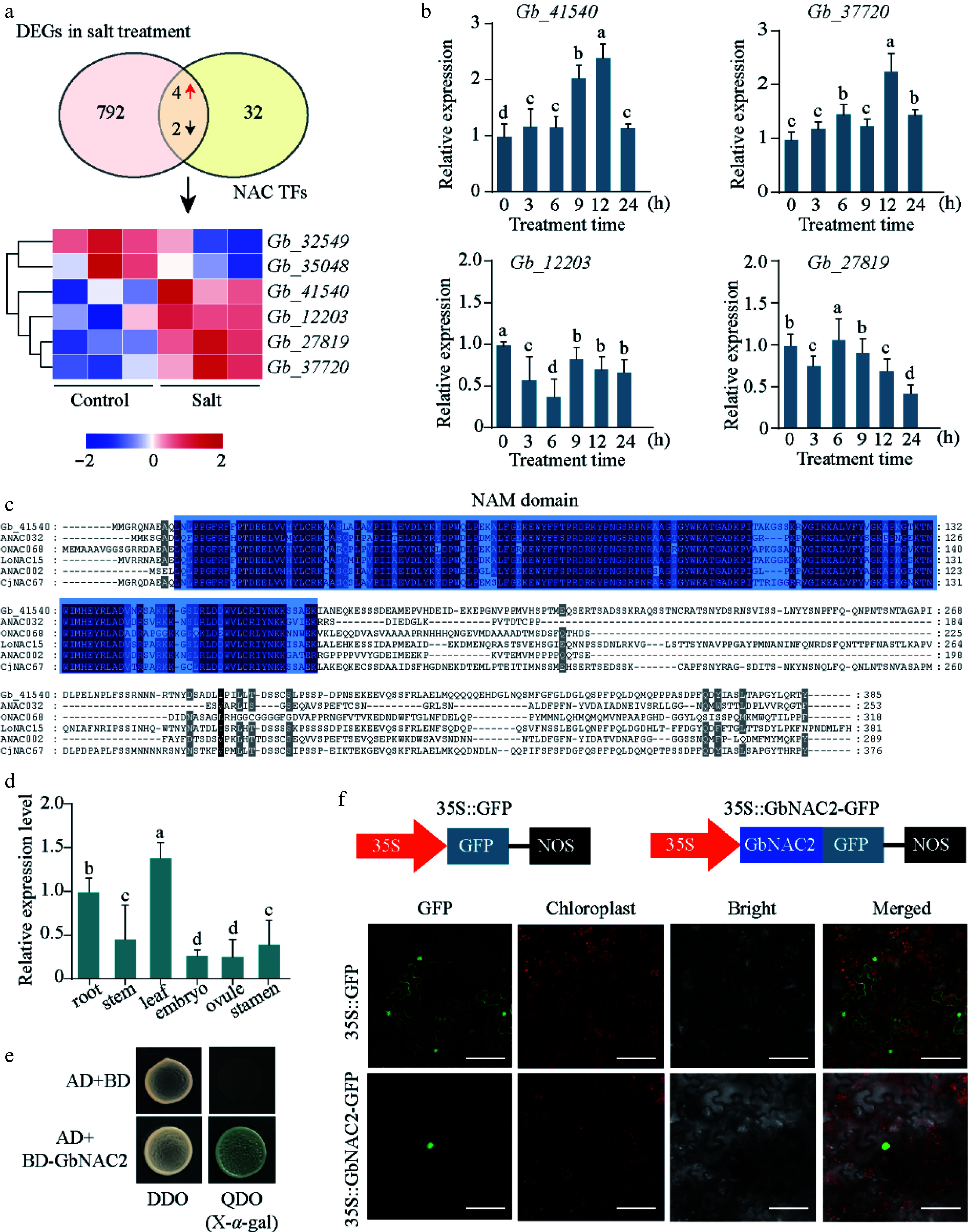
Identification of key NAC TFs. (a) Overlap analysis of *NAC* genes in *G. biloba* and DEGs under salt stress transcriptome. The FPKM value of six key *NACs* following salt treatment are depicted in a heatmap (red represents upregulated, and blue represents downregulated). (b) The relative expression of four *NAC* genes at different time points following salt treatment was quantified using qPCR. (c) Sequence analysis of *GbNAC2* and other NAC TFs from different plant species. (d) The spatial-temporal expression of *GbNAC2* in different issues in ginkgo. (e) Analysis of transcriptional activation activity of GbNAC2. (f) Subcellular localization of the GbNAC2 protein. Scale bar = 10 μm. Different letters indicate significant differences (one-way analysis of variance, *p* < 0.05).

A previous study has shown that *NAC* genes in ginkgo exhibit significant responses to salt stress^[13]^. In this study, four *NAC* genes positively responsive to salt stress were identified from RNA-seq data of salt-treated ginkgo leaves (Fig. 1a). To determine which NAC genes participate in the salt stress response in Ginkgo, seedlings were treated with 200 mM NaCl. The expression patterns of the four salt-induced NAC genes were analyzed by qRT-PCR within the first 24 h following treatment. Among them, *Gb_41540* showed a rapid induction within 3 h and maintained elevated expression levels in the leaves for up to 24 h (Fig. 1b).

*Gb_41540* contains an open reading frame of 1,158 bp and is predicted to encode a 385-amino-acid protein featuring a conserved NAM domain typical of the NAC transcription factor family (Fig. 1c). Phylogenetic analysis showed that the Gb_41540 protein was clustered together with NAC002 from *A. thaliana*, and Gb_41540 was referred to as GbNAC2 (Supplementary Fig. S1). Tissue-specific expression analysis revealed that the highest *GbNAC2* expression in leaves and roots (Fig. 1d). To test the transcriptional activation activity of the GbNAC2 protein, the full-length GbNAC2 was fused to the GAL4 DNA-binding domain of the pGBKT7 (BD) vector and transformed into yeast. The yeast's growth and blue color on quadruple-dropout medium with X-*α*-gal indicated GbNAC2 has transcriptional activation (Fig. 1e). Additionally, the transient transformation of tobacco confirmed the nuclear localization of GbNAC2 (Fig. 1f).

### Overexpressing *GbNAC2* promotes root development

**Figure 2:**
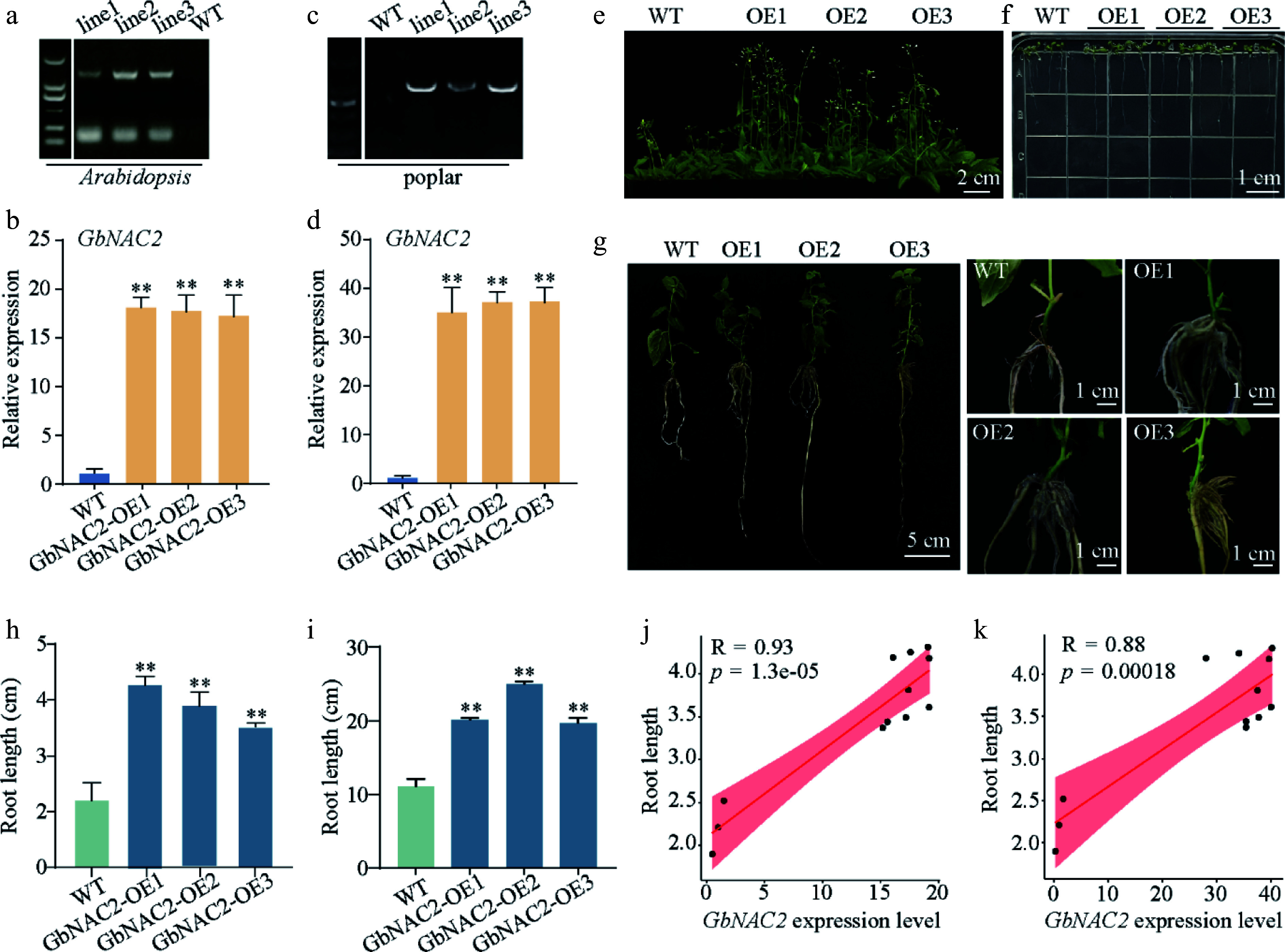
Phenotypic analysis of overexpressed *GbNAC2* in *Arabidopsis* and poplar. The (a), (c) positive detection and (b), (d) *GbNAC2* expression level in transgenic plants. Ectopic expression of *GbNAC2* significantly promotes the (e) flowering, and (f) root development of WT *Arabidopsis*. (g) The ectopic expression of *GbNAC2* markedly enhances the development of primary and lateral roots in poplar. A statistical analysis of the root length of (h) transgenic *Arabidopsis,* and (i) transgenic poplar was conducted. The relationship between *GbNAC2* expression and root length in (j) transgenic *Arabidopsis,* and (k) poplar. ** *p* < 0.01.

To explore the functional role of *GbNAC2*, *GbNAC2* overexpression *Arabidopsis* transgenic lines (*GbNAC2*-OE) were generated (Fig. 2a, d). The *GbNAC2*-OE *Arabidopsis* displayed accelerated growth and earlier flowering phenotype compared to the WT (Fig. 2e). Additionally, transgenic *Arabidopsis* roots were 1.6–2.0-fold longer than those of the WT plants (Fig. 2f, h). *GbNAC2*-OE transgenic poplar lines were also generated (Fig. 2c, d). In consistent with *GbNAC2*-OE *Arabidopsis* lines, compared to the WT, the number of lateral roots in *GbNAC2*-OE poplar plants was significantly increased, and root length was approximately 1.6- to 2.4-fold longer than in WT plants (Fig. 2g, i). Furthermore, correlation analysis results displayed a strong association between the expression level of *GbNAC2* and root length in transgenic plants (Fig. 2j, k).

### GbNAC2 enhances salt tolerance by inhibiting ROS accumulation

**Figure 3:**
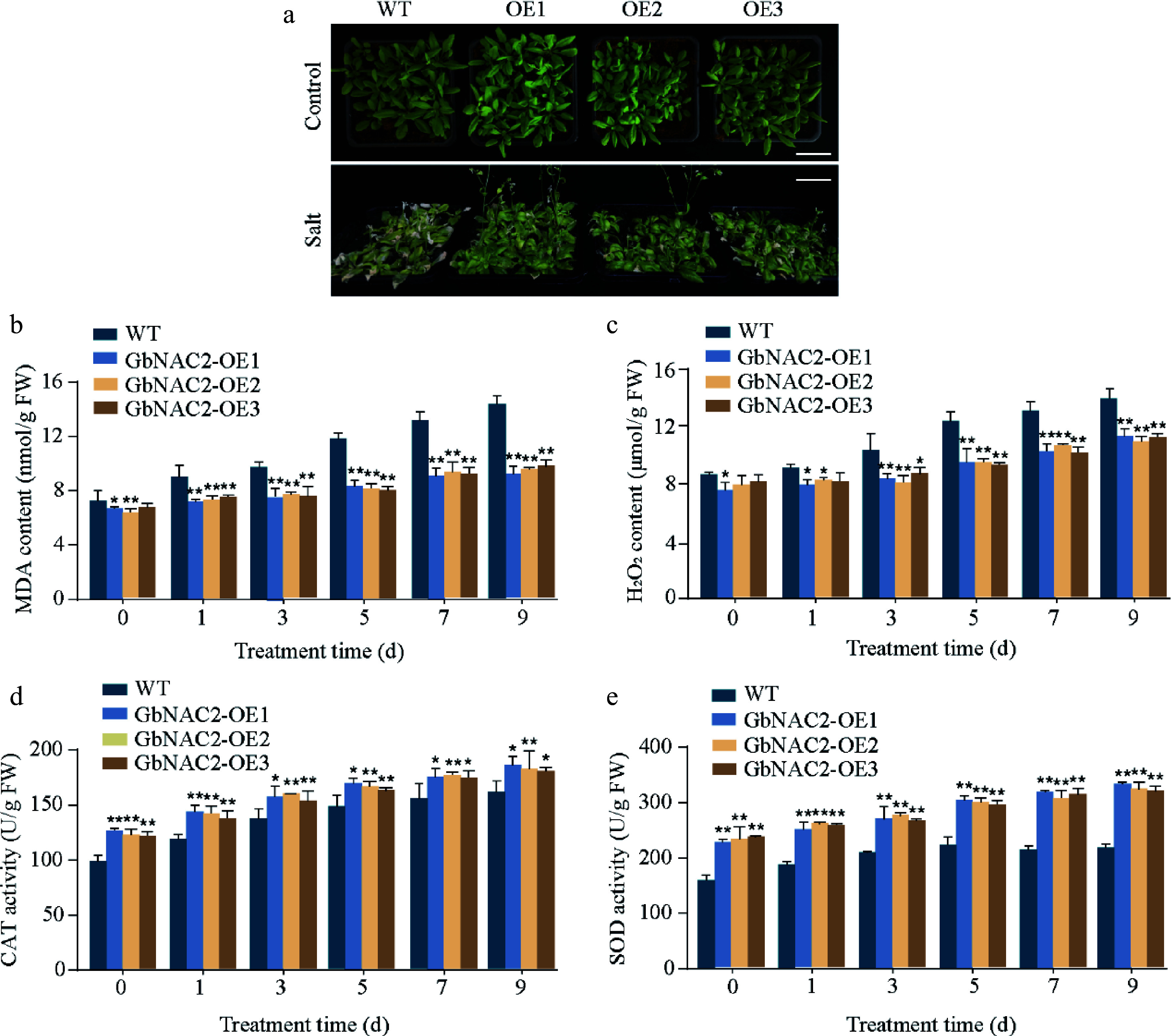
Overexpression of *GbNAC2* enhances the salt tolerance in *Arabidopsis*. (a) Phenotypic appearance of the plants after 9 d of salt treatment. (b) Levels of MDA in leaves. (c) Levels of H_2_O_2_ in leaves. (d) Activities of CAT in leaves. (e) Activities of SOD in leaves. * *p* < 0.05, ** *p* < 0.01.

To assess the role of *GbNAC2* in salt stress tolerance, WT plants showed pronounced leaf wilting and bleaching under salt stress, whereas *GbNAC2*-OE lines exhibited much milder chlorosis (Fig. 3a). The MDA content, which is a key parameter of stress tolerance, within these plants was further examined^[25]^. After salt treatments, the accumulation of MDA in WT plants was significantly higher than that in the *GbNAC2*-OE plants (Fig. 3b). Additionally, salt stress also increased the content of H_2_O_2_ in all plants, but the increase rate was less in *GbNAC2*-OE lines compared to WT (Fig. 3c). Antioxidant enzyme activities were also measured, finding that both CAT and SOD activities were significantly higher in the *GbNAC2*-OE lines than in WT plants (Fig. 3d, e). Germination assays showed no difference between WT and transgenic *Arabidopsis* under normal conditions, but under salt stress, transgenic seeds displayed significantly higher germination rates (Supplementary Fig. S2).

**Figure 4:**
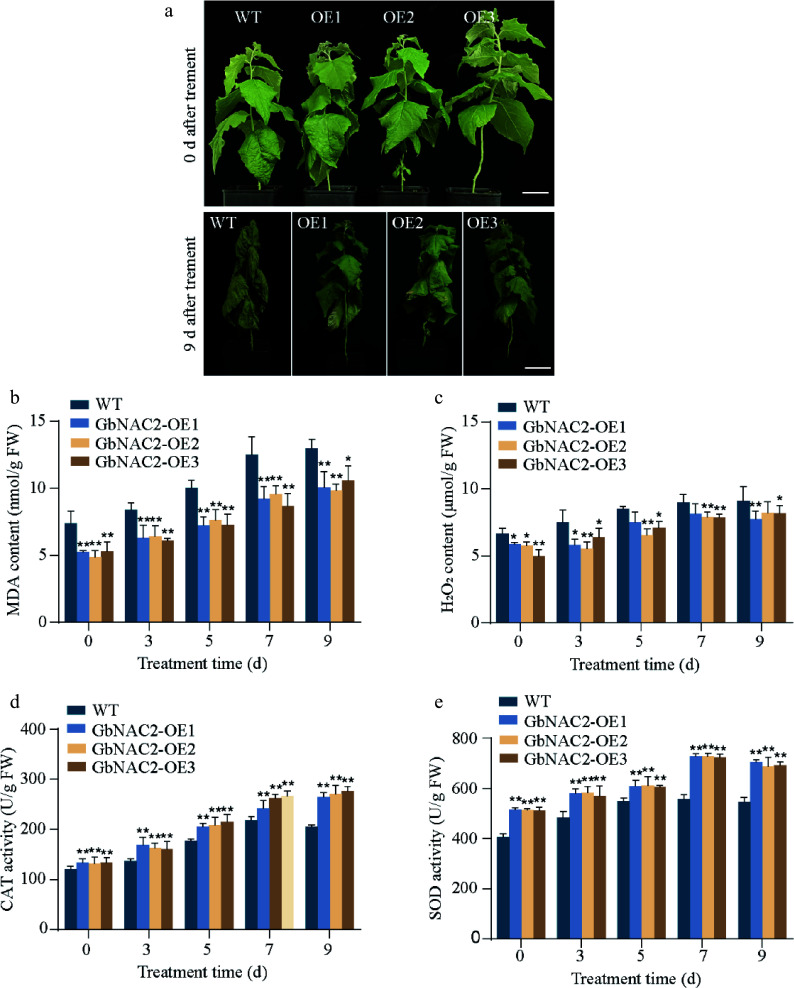
Overexpression of *GbNAC2* enhances the salt tolerance in poplar. (a) Phenotypic appearance of the plants under salt treatment. (b) Levels of MDA in leaves. (c) Levels of H_2_O_2_ in leaves. (d) Activities of CAT in leaves. (e) Activities of SOD in leaves. * *p* < 0.05, ** *p* < 0.01.

Similar results were observed in *GbNAC2*-OE poplar. WT poplars showed noticeable leaf yellowing after 5 d of salt treatment, with most leaves wilting and dying by day 9. In contrast, *GbNAC2*-OE lines maintained healthy growth throughout the treatment period (Fig. 4a). Physiological analyses under salt stress revealed that *GbNAC2*-OE poplars consistently had lower levels of H_2_O_2_ and MDA, along with higher CAT and SOD activities compared to WT plants (Fig. 4b, e). Together, these findings suggest that *GbNAC2* significantly enhances salt tolerance by reducing oxidative stress and boosting antioxidant enzyme activity, leading to improved growth and survival under salt stress conditions.

### *GbNAC2* promotes flavonoid biosynthesis

**Figure 5:**
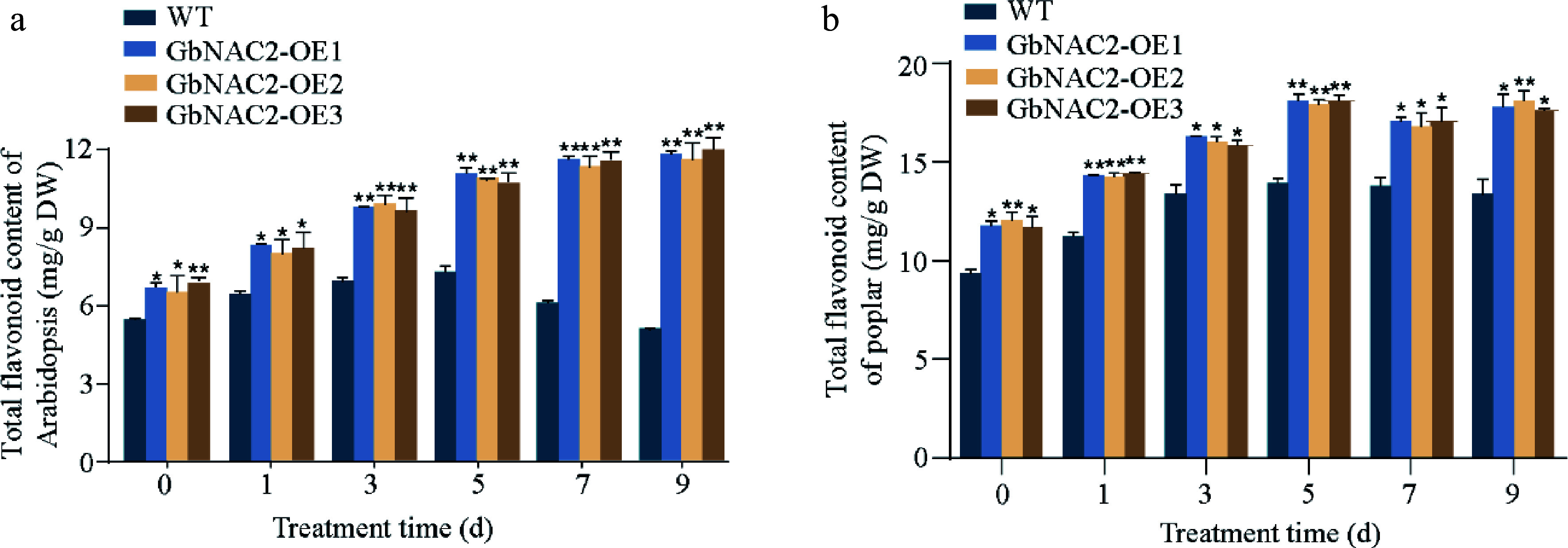
Determination of flavonoid content at different time points following salt treatment in transgenic (a) *Arabidopsis,* and (b) transgenic poplar. * *p* < 0.05, ** *p* < 0.01.

The flavonoid content in *GbNAC2*-OE lines after salt treatment were measured. Prior to treatment, the flavonoid content in transgenic *Arabidopsis* and poplar were 1.2- and 1.3-fold that of the WT, respectively. During salt stress, flavonoid content in WT plants initially increased but then declined, whereas in *GbNAC2*-OE *Arabidopsis*, flavonoid levels steadily rose throughout the treatment. After 9 d of salt exposure, flavonoid content in the transgenic *Arabidopsis* was about 2.1 times higher than in WT plants (Fig. 5a). A similar pattern was observed in poplar, with flavonoid levels in *GbNAC2*-OE lines reaching approximately 1.4-fold that of WT after 9 d (Fig. 5b).

**Figure 6:**
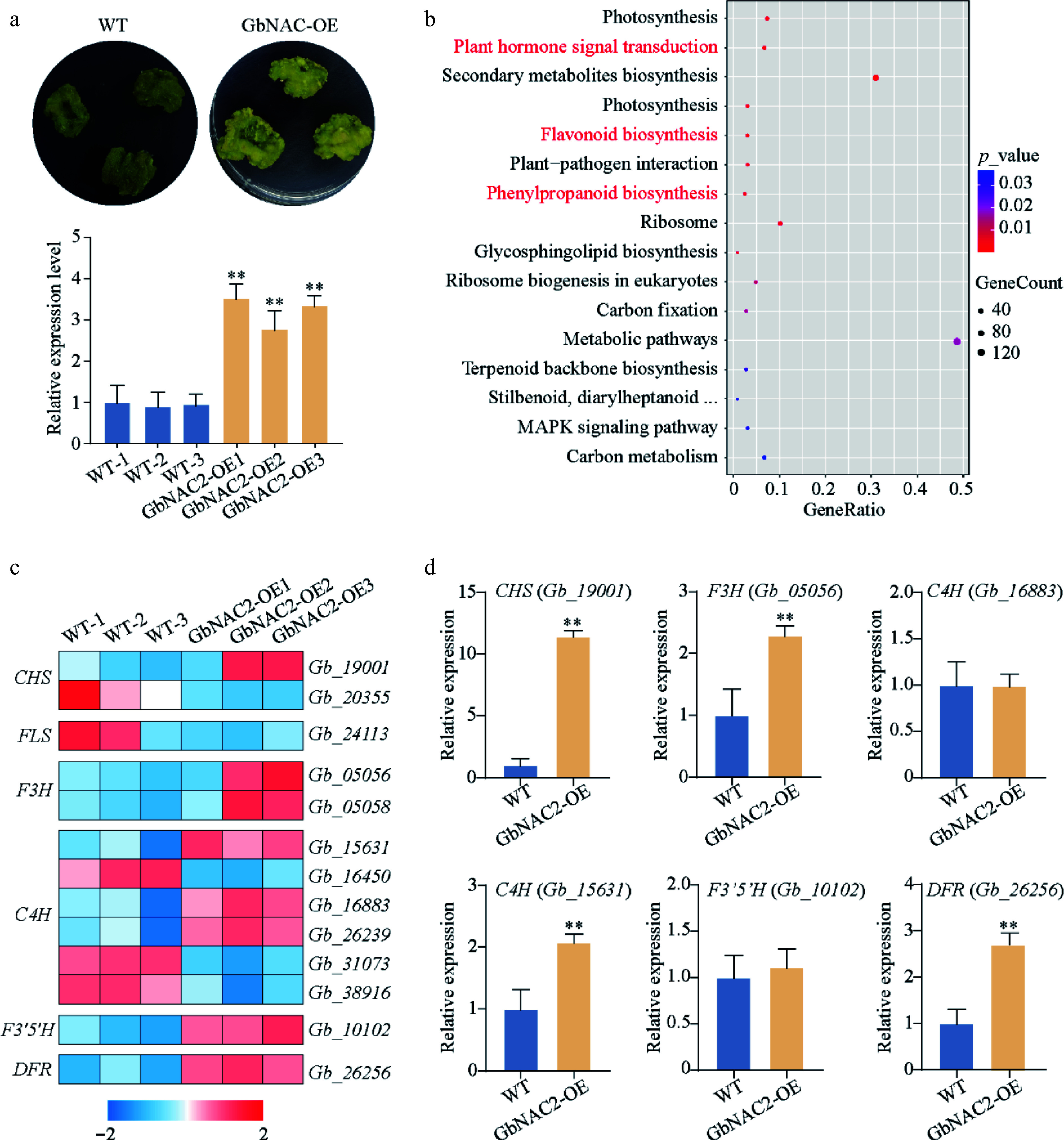
Transcriptome analysis reveals downstream signaling pathways regulated by *GbNAC2*. (a) Expression of *GbNAC2* in *GbNAC2*-OE ginkgo calli. (b) Enrichment analysis of DEGs in the KEGG pathways. (c) A heatmap is presented to illustrate the transcriptional changes of genes in the flavonoid pathway between WT and transgenic calli (red represents upregulated, and blue represents downregulated). (d) qPCR analysis of key structural genes. ** *p* < 0.01.

To elucidate the molecular mechanisms how *GbNAC2*-mediated salt response, *GbNAC2* was overexpressed in ginkgo calli, followed by transcriptomic analysis. The expression level of *GbNAC2* in the calli was significantly higher compared to non-transformed samples, suggesting successful overexpression of *GbNAC2* in calli (Fig. 6a). A total of 2,043 differentially expressed genes (DEGs) were identified between the *GbNAC2*-OE calli and control, with 1,059 genes upregulated, and 984 genes downregulated (Supplementary Fig. S3). KEGG enrichment analysis demonstrated significant enrichment of these DEGs in phenylpropanoid biosynthesis, flavonoid biosynthesis, and plant hormone signal transduction pathways (Fig. 6b). Given that the overexpression of *GbNAC2* significantly promotes the synthesis of flavonoids, key genes related to the flavonoid biosynthesis pathway were focused on. Critical structural genes involved in the flavonoid biosynthesis pathway exhibited coordinated upregulation, including *cinnamate 4-hydroxylase* (*C4H*), *chalcone synthase* (*CHS*), *flavanone 3-hydroxylase* (*F3H*), *flavonoid 3′,5′-hydroxylase* (*F3′5′H*), and *dihydroflavonol 4-reductase* (*DFR*) (Fig. 6c). These transcriptional changes were validated by qRT-PCR, which confirmed consistent upregulation patterns (Fig. 6d). These results suggest that *GbNAC2* functions as a regulatory hub to amplify flavonoid biosynthesis by modulating the expression of key flavonoid biosynthesis pathway genes under salt stress.

### Identification of genes involved in hormone signaling pathways

Systematic analysis of hormone-related DEGs in *GbNAC2*-OE calli was performed to identify the hormone signaling networks influenced by *GbNAC2*. Intriguingly, 12 DEGs were significantly enriched in the auxin signaling pathway, including five *SAUR* (*SMALL AUXIN UP-REGULATED RNA*) genes—a class of early auxin-responsive regulators implicated in cell expansion and root development^[26]^. Notably, all differentially expressed *SAUR* genes exhibited pronounced upregulation in transgenic calli, with qRT-PCR confirming the elevated expression of five representative *SAUR* paralogs (Supplementary Fig. S4).

**Figure 7:**
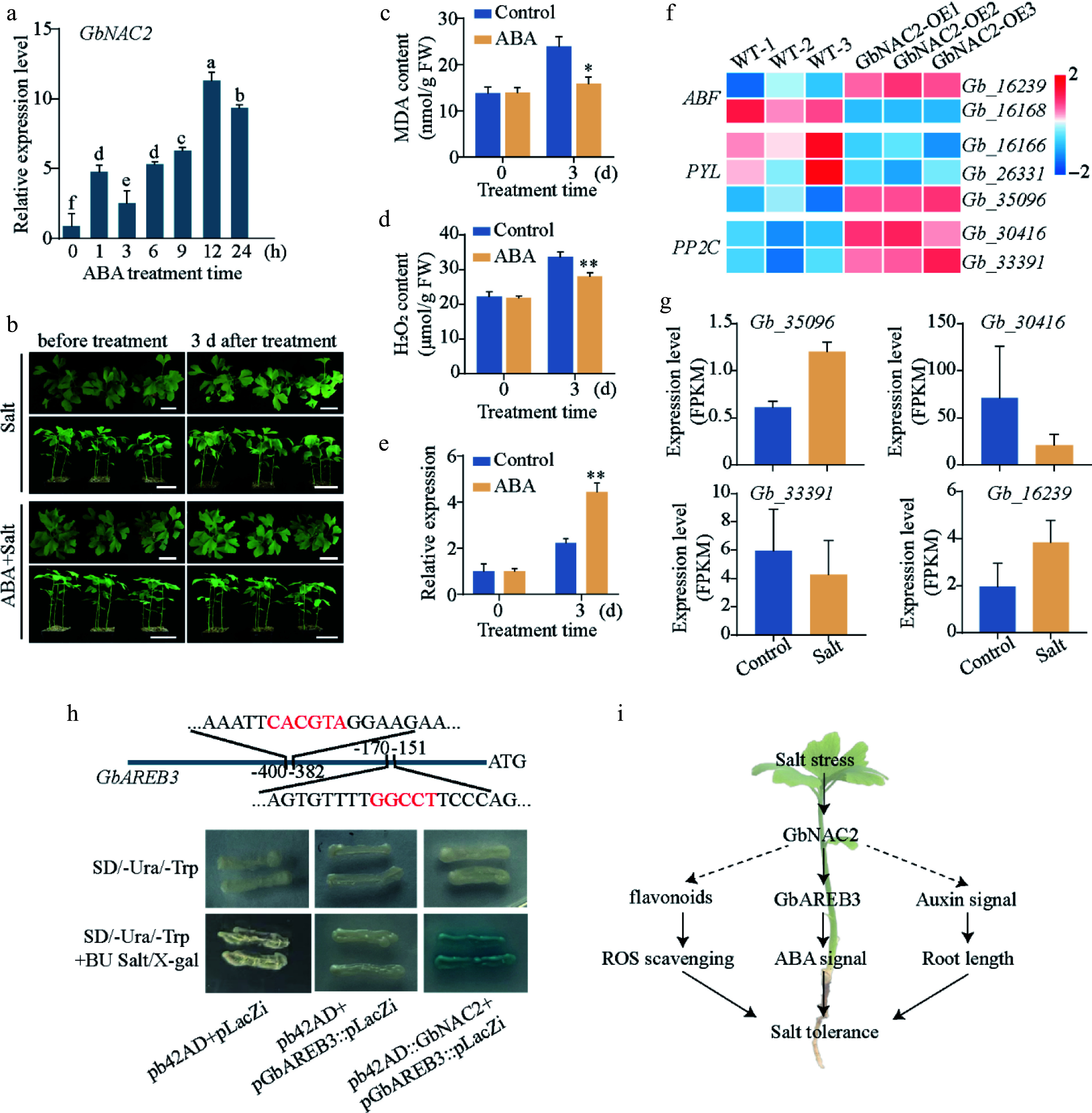
GbNAC2 participates in ABA signaling by interacting with *GbAREB3* promoters. (a) The expression profile of *GbNAC2* within 24 h post ABA treatment. Different letters indicate significant differences (one-way analysis of variance, *p* < 0.05). (b) Phenotypic changes in ginkgo seedlings before and after NaCl and ABA + NaCl treatment. Scale bar = 4 cm. The content of (c) MDA, (d) and H_2_O_2_ before and after NaCl and ABA + NaCl treatment. (e) The expression of *GbNAC2* before and after treatment. * *p* < 0.05, ** *p* < 0.01. (f) Heatmap of significantly differentially expressed ABA pathway-related genes following overexpression of *GbNAC2*. (g) Expression analysis of four ABA pathway-related genes under salt stress. (h) Verification of the interaction between GbNAC2 and the *GbAREB3* promoter through Y1H assay. (i) Schematic diagram illustrating the promotion of salt stress tolerance in ginkgo by *GbNAC2*.

ABA signaling has been widely documented to play a crucial role in regulating plant salt tolerance^[27]^. To explore ABA's role in ginkgo salt response, *GbNAC2* transcription was analyzed in ABA-treated seedlings. The results showed that exogenous ABA significantly induced the *GbNAC2* expression, with rapid upregulation within 1 h, and reaching a peak at 12 h (Fig. 7a). Ginkgo seedlings were then treated with 400 mM NaCl alone or in combination with 20 μM ABA. Severe wilting phenotypes in NaCl-treated plants were found whereas the ABA-treated plants maintained good growth status after salt treatment, with only minor leaf wilting (Fig. 7b). After 3 d of salt treatment, the seedlings co-treated with exogenous ABA and NaCl exhibited significantly lower levels of MDA and H_2_O_2_ compared to those treated with NaCl alone (Fig. 7c, d). Meanwhile, the expression level of *GbNAC2* increased by more than 2-fold after salt treatment; under the co-treatment of ABA and salt stress, its expression level further increased by more than 4-fold (Fig. 7e). In the *GbNAC2*-OE ginkgo calli, seven genes related to the ABA signaling pathway were identified, among which four genes were up-regulated (Fig. 7f). Notably, the genes *Gb_35096* and *Gb_16239* exhibited positive responses to salt stress (Fig. 7g).

### GbNAC2 activates GbAREB3 expression by directly binding to its promoter

Among those candidates, two NAC family protein-binding SNBE elements were identified on the GbAREB3 promoter, located at 151–170 and 382–400 bp, respectively. Through evolutionary analysis with *Arabidopsis*, Gb_16239 is most closely related to AtAREB3. Consequently, *Gb_16239* has been designated as *GbAREB3* (Supplementary Fig. S5). It is hypothesized that *GbAREB3* is regulated upstream by NAC factors. To investigate the binding of GbNAC2 to the *GbAREB3* promoter, the Y1H assay was utilized to confirm that GbNAC2 can directly bind to the *GbAREB3* promoter. The results showed that yeast cells containing both GbNAC2 and *pGbAREB3* exhibited normal growth and turned blue on a selective medium containing BU salt and X-gal. In contrast, yeast cells containing only *pGbAREB3* were able to grow but did not turn blue on the same selective medium (Fig. 7h). These results suggest that GbNAC2 can directly bind to the *GbAREB3* promoter to activate its expression.

## Discussion

*G. biloba* leaves are rich in flavonoids, which serve as essential raw materials for the pharmaceutical and nutraceutical industries^[28]^. Salt stress poses a significant challenge to plant growth, severely impacting ginkgo trees. However, the molecular mechanisms underlying ginkgo's tolerance to salt stress are still largely unexplored. NAC TFs are one of the largest gene families in plants and have been widely reported as pivotal in plant responses to salinity^[29]^. In this study, qRT-PCR results indicated that *GbNAC2* responds rapidly to salt treatments with a significant increase in transcriptional levels, suggesting its potential role as a key regulator of ginkgo's salt stress tolerance. Notably, *GbNAC2* is predominantly expressed in leaves and roots. To further investigate its function, *GbNAC2* was overexpressed in both *Arabidopsis* and poplar. Compared to control plants, the transgenic plants exhibited significantly enhanced growth under salt stress, demonstrating improved salt tolerance. These data validate the vital role of *GbNAC2* in enhancing ginkgo's salt stress resilience.

In the physiological regulatory network of plants adapting to the environment, the trade-off between growth and stress resistance is a universally existing core strategy. Due to the limited availability of resources such as carbon sources and energy in plants, the enhancement of resistance to external stresses is often accompanied by reduced growth^[30]^. This phenomenon has been repeatedly verified in studies on the regulation of plant stress resistance by NAC transcription factors. For instance, in *Populus euphratica*, *PeNAC036*-overexpressing plants exhibit significantly inhibited growth and enhanced salt tolerance^[31]^. In *Populus euphratica*, the overexpression of the *PeNAC122* inhibits plant growth while significantly enhancing its resistance to osmotic stress^[32]^. These examples demonstrate the typical trade-off relationship of stress resistance enhancement coupled with growth inhibition. Nevertheless, some studies have found that certain *NAC* genes can break through this trade-off constraint. For example, overexpression of *BpNAC2* from *Betula platyphylla* not only promotes plant growth but also enhances adaptability to salt stress^[33]^. *PtNAC3* from *Pinus tabuliformis* not only enhances the resistance of transgenic plants to multiple abiotic stresses, but also promotes the seed yield of plants under stress conditions^[34]^.

In this study, it was found that overexpression of *GbNAC2* promoted the growth and salt resistance of transgenic plants. Meanwhile, the root length of transgenic plants was increased by more than 1.6-fold compared with the control. Transcriptome analysis revealed that the expression level of the auxin-responsive gene *SAUR* was upregulated by more than 2-fold in *GbNAC2*-overexpressing plants relative to the control. As downstream effectors of the auxin signal, *SAUR* family genes can promote the elongation of cells in the root elongation zone by activating plasma membrane H^+^-ATPase^[35]^. Considering that roots serve as the primary organ for plants to absorb water and perceive stress signals, the increase in root length can expand the water absorption range and reduce the accumulation of salt ions in the root epidermis^[36]^. Therefore, it is proposed that *GbNAC2* may regulate the transcriptional expression of *SAUR* to initiate auxin-mediated root development, thereby enhancing salt stress tolerance. However, the precise regulatory mechanism between *GbNAC2* and *SAUR* requires further clarification.

Salt stress triggers ionic and osmotic imbalances, which can lead to secondary stresses, particularly oxidative stress caused by ROS. Consequently, maintaining homeostatic levels of ROS through scavenging pathways is crucial for protecting plants from oxidative damage^[37]^. Plants predominantly utilize protective strategies to scavenge ROS: enzymatic and non-enzymatic antioxidants^[38]^. SOD and CAT are two crucial enzymes within the enzymatic detoxification system^[39]^. In this study, measurements of H_2_O_2_ content revealed that overexpression of *GbNAC2* suppresses ROS accumulation induced by salt treatments. The levels of SOD and CAT were significantly higher in the *GbNAC2*-OE lines than in the WT plants. These findings indicate that *GbNAC2* may regulate ginkgo salt tolerance through ROS scavenging.

In addition to the enzymatic metabolic system, numerous metabolites function as non-enzymatic antioxidants^[40]^. Flavonoids are particularly important in this context and have been shown to possess antioxidant properties^[41]^. Extensive research has highlighted the significant role of flavonoids in mitigating the adverse impacts of salt stress by increasing the levels of antioxidant enzymes and reducing ROS accumulation^[42,43]^. Gao et al.^[44] ^found that salt stress induces the expression of *EbbHLH80*, and overexpression of *EbbHLH80* enhances salt tolerance, accompanied by elevated flavonoid accumulation and reduced ROS levels compared to WT plants. Moreover, the IAA17.1/HSFA5a module modulates poplar root adaptation to salt stress by regulating flavonol biosynthesis and controlling ROS accumulation^[45]^. The present findings demonstrated that overexpression of *GbNAC2* facilitates the accumulation of flavonoids and activates genes associated with flavonoid biosynthesis, including *CHS*, *F3H*, *C4H*, and *DFR*. Notably, *GbNAC2*-OE plants exhibited an increasing trend in flavonoid accumulation after salt treatments. These results suggest that *GbNAC2* may enhance ginkgo's salt tolerance by regulating flavonoid biosynthesis to maintain ROS homeostasis. However, whether *GbNAC2* directly regulates these flavonoid biosynthesis genes requires further investigation.

Plant hormones play a critical role in regulating plant salt tolerance, with ABA being a key hormone in modulating the plant's response to salt stress. Under high salinity, ABA mediates stomatal closure, thereby preventing water loss due to osmotic stress^[8,46]^. Exogenous ABA treatment has been shown to enhance the salt tolerance of rice seedlings^[47]^, while the rice *SAE1* gene influences salt stress tolerance by inhibiting ABI5-mediated ABA signaling^[48]^. Conversely, *PalWRKY77* in poplar mitigates ABA-mediated growth inhibition and stomatal closure by suppressing the expression of ABA-responsive genes, thereby reducing salt tolerance^[49]^. In this study, after three days of salt treatment, ginkgo seedlings exhibited a wilting phenotype, and concurrently, the expression level of *GbNAC2* was significantly upregulated. Furthermore, it was observed that the application of exogenous ABA could significantly alleviate the damage caused by high salt stress to ginkgo seedlings, with the expression level of *GbNAC2* increasing by more than 4-fold. These results indicate that elevated *GbNAC2* expression contributes to alleviating the damage caused by salt stress in ginkgo. A key *GbAREB3* gene was also identified in the ABA signaling pathway downstream of GbNAC2. GbAREB3 belongs to the ABF family of transcription factors, which are widely reported to be involved in the plant stress response^[50]^. It was found that the *GbAREB3* transcription level is regulated by salt stress, and overexpression of *GbNAC2* promotes the *GbAREB3* expression. Y1H validation demonstrated that GbNAC2 can directly bind to the *GbAREB3* promoter to regulate its expression. Therefore, *GbNAC2* enhances ginkgo's salt stress tolerance by regulating *GbAREB3* expression to activate the ABA signaling pathway.

## Conclusions

In conclusion, the present study elucidates the dual role of *GbNAC2* in enhancing salt tolerance in ginkgo through the integrated regulation of flavonoid biosynthesis and auxin-ABA crosstalk (Fig. 7i). As a central transcriptional regulator, *GbNAC2* promotes stress-resilient root architecture remodeling and flavonoid-driven antioxidant capacity by activating auxin signaling and flavonoid metabolic genes. Simultaneously, it amplifies ABA-mediated detoxification through direct binding to the *GbAREB3* promoter. This unique coordination of developmental plasticity (via auxin), and stress-responsive metabolism (via flavonoids/ABA signaling) effectively resolves the growth-defense trade-off under salinity stress. Together, these results demonstrate that *GbNAC2* serves as a key salt-tolerance gene in ginkgo, contributing to salinity adaptation through multiple avenues. Its multifunctional role makes *GbNAC2* a promising target for biotechnological strategies aimed at improving salt tolerance in ginkgo through molecular breeding.

## SUPPLEMENTARY DATA

Supplementary data to this article can be found online.

## Data Availability

The raw data of the transcriptome in this study are stored in the National Genomics Data Center's Genome Sequence Archive, with Accession No. CRA016156.
