# Supplementary material for: Dual function of GbNAC2 in flavonoid metabolism and hormonal pathways enhances salt tolerance in Ginkgo biloba
Source: For Res (Fayettev). 2025 Nov 20;5:e028. doi: 10.48130/forres-0025-0027 (PMC12648015; doi:10.48130/forres-0025-0027)
Supplement: Supplementary file 1 — Supplementary data to this article can be found online. [file FR-2025-5-0027-Supplementary.zip › 10.48130_forres-0025-0027-Suppl-FigureS2.pdf]

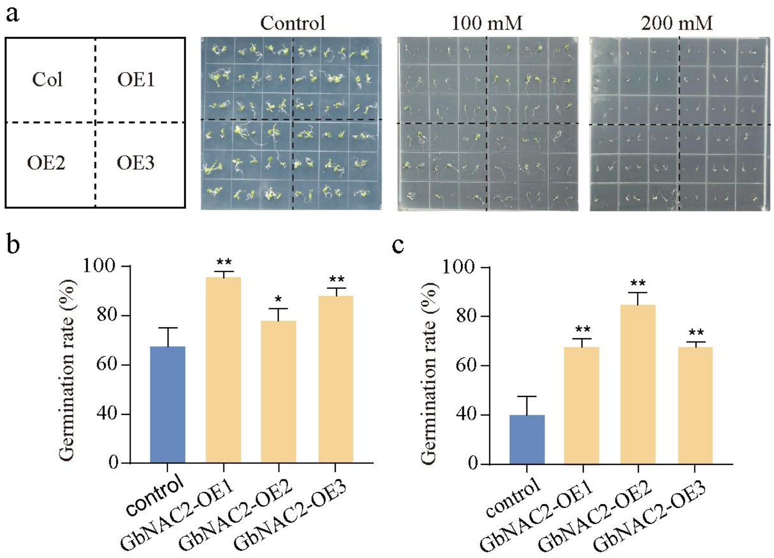

Figure S2. The Effect of *GbNAC2* overexpression on seed germination in transgenic *Arabidopsis*. **(a)** The germination status of *Arabidopsis* seeds under treatment with different concentrations of NaCl. Statistics of germination rate under treatment with 100mM **(b)** and 200mM **(c)** NaCl. Data are means ± SD (n = 3). *P < 0.05, **P < 0.01.
