# Supplementary material for: Dual function of GbNAC2 in flavonoid metabolism and hormonal pathways enhances salt tolerance in Ginkgo biloba
Source: For Res (Fayettev). 2025 Nov 20;5:e028. doi: 10.48130/forres-0025-0027 (PMC12648015; doi:10.48130/forres-0025-0027)
Supplement: Supplementary file 1 — Supplementary data to this article can be found online. [file FR-2025-5-0027-Supplementary.zip › 10.48130_forres-0025-0027-Suppl-FigureS4.pdf]

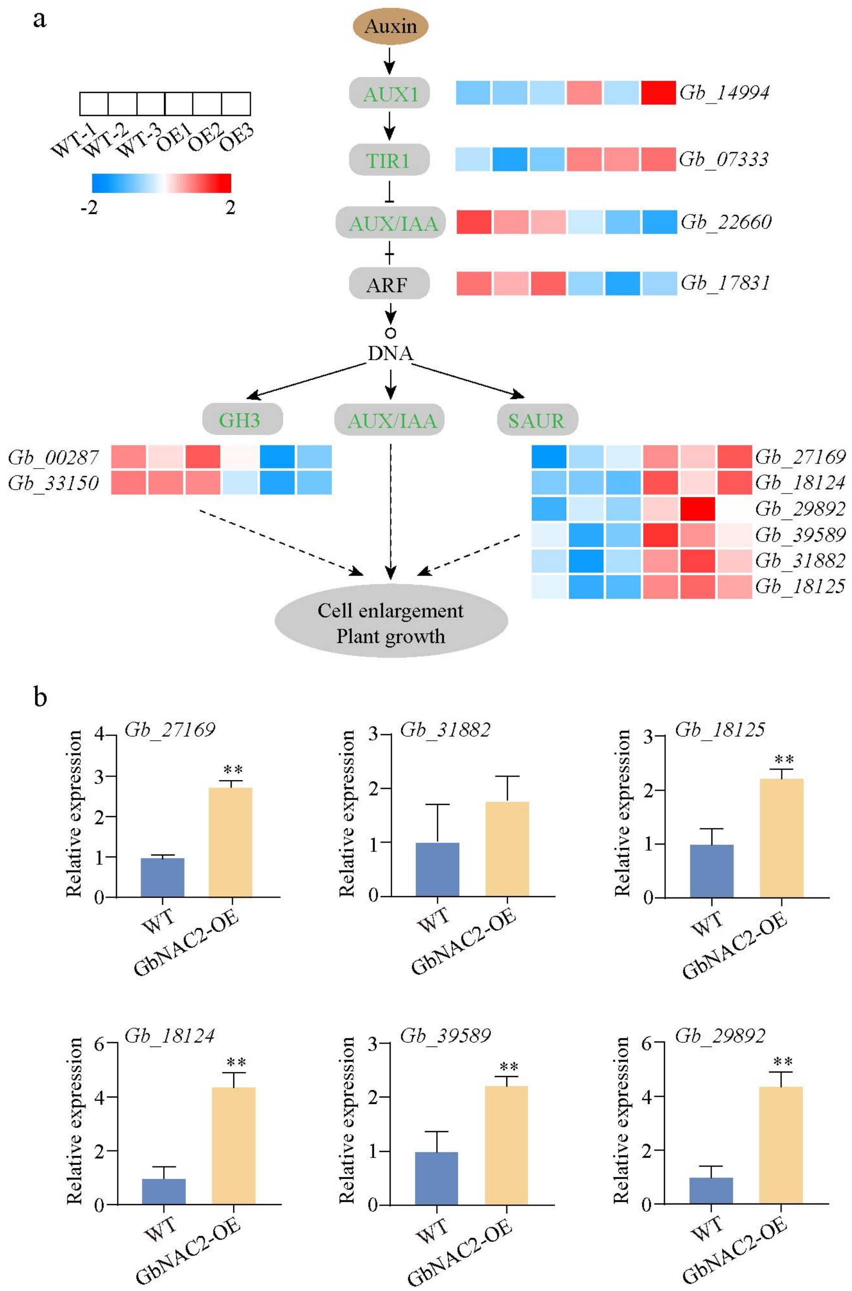

Figure S4. Analysis of genes related to Auxin pathway. **(a)** A heatmap is presented to illustrate the transcriptional changes of genes in the flavonoid pathway between WT and transgenic calli (red represents upregulated and blue represents downregulated). **(b)** qPCR analysis of key structural genes. Data are means ± SD (n = 3). $^{**}P < 0.01$.
