# Supplementary material for: Dual function of GbNAC2 in flavonoid metabolism and hormonal pathways enhances salt tolerance in Ginkgo biloba
Source: For Res (Fayettev). 2025 Nov 20;5:e028. doi: 10.48130/forres-0025-0027 (PMC12648015; doi:10.48130/forres-0025-0027)
Supplement: Supplementary file 1 — Supplementary data to this article can be found online. [file FR-2025-5-0027-Supplementary.zip › 10.48130_forres-0025-0027-Suppl-TableS1.pdf]

Table S1. Names and corresponding accession numbers (IDs) of genes used in this work.

	Gene Name	Accession number or Gene ID
**Sequence analysis**	ANAC032	Q9CAR0.1
	ONAC068	Q52QH4.1
	LoNAC15	WLM68367.1
	ANAC002	Q39013.2
	CjNAC67	XP_057859547.2
**Phylogenetic analysis**	AtABF1	AT1G49720
	AtAREB2	AT3G19290
	AtABF3	AT4G34000
	AtDPBF4	AT2G41070
	AtAREB3	AT3G56850
	AtABI5	AT2G36270
	AtDPBF2	AT3G44460
	AtAREB1	AT1G45249
**Main genes in this work**	GbNAC2	Gb_41540
	GbAREB3	Gb_16239
