# Supplementary material for: Dual function of GbNAC2 in flavonoid metabolism and hormonal pathways enhances salt tolerance in Ginkgo biloba
Source: For Res (Fayettev). 2025 Nov 20;5:e028. doi: 10.48130/forres-0025-0027 (PMC12648015; doi:10.48130/forres-0025-0027)
Supplement: Supplementary file 1 — Supplementary data to this article can be found online. [file FR-2025-5-0027-Supplementary.zip › 10.48130_forres-0025-0027-Suppl-TableS2.pdf]

Table S2. Oligonucleotide sequences of the primers used in this work.

	Sequence (5' to 3')		
	**Gene Name**	**Forward primers**	**Reverse primers**
**35S::GFP**	**GbNAC2**	tccagctccaggatccATGATGGGAAGACAGAATGCAGAAG	gagaaagcttggatccATATGTCCTTTGTAAGTATCCTGGA
**Transcriptional activation activity**	**GbNAC2-BD**	aggccgaattcccggggatccATGATGGGAAGACAGAATGCAGAAG	ccgctgcaggtcgacggatccATATGTCCTTTGTAAGTATCCTGGA
**Y1H assays**	**GbNAC2-pb42AD**	gattatgcctctcccgaattcATGATGGGAAGACAGAATGCAGAA[illegible]	agaagtccaaagcttctcgagATATGTCCTTTGTAAGTATCCTGGA
	**pGbAREB3-pLacZi**	cttgaattcgagctcggtaccCATCATCTTTTGCCTTGAGAAGC	agcacatgcctcgaggtcgacTTGATTCAATCCAGAAGCTTATATC
**RT-qPCR**	**GbNAC2**	AAGGGAACCAAGACTAATTGGATTAT	CTGCTCATTTGCTATCTTTTCTGC
	**Gb_37720**	ATAAACCGAATAACCGCCTCC	CCTGTACTCATTCATTATCCAAT
	**Gb_27819**	ATCTGGATCAATTCATGGTCGCC	TTGCATGCATGGTTGCTTGAA
	**Gb_12203**	AGGTTCAGAATGGAGGCCGTCCT	AATCAGTCTTCTGCCCTCGTG
	**Gb_19001**	GCTGGGCGGACTGGAAACATC	CACTCTGCGATTCTTGCCAACAAC
	**Gb_05056**	GCGTGAGGGAGGAGGTGAAGG	CTGGCGGGAGGGCAAAGAAATC
	**Gb_16883**	AACTTAGCTAAGAAATATGGAGAT	GCAGAACGATATCTCCATATTT
	**Gb_15631**	ATGTCAGTGATGGGCAGTTGTTCC	TCCAGGTGGAGCAGGTGAAGTAG
	**Gb_10102**	CTTGGGCGGGAAAGCGTTGG	GTTGGAGCGGCGAGAACTGTG
	**Gb_26256**	AACCCTGCGGTGAAAGGAGTTG	AAGCTGCGGAAAGCGAGGAAG
	**Gb_27169**	TTTATGTCGGGCAGGAACGCAAG	CCTTCTCAGCCTCGTCCAAAAGC
	**Gb_31882**	CGATCCCGTGACCAGTTCTTGC	CCGCTTCTGCTCTTCGCCAAC
	**Gb_18125**	AGAACTCGGCTCGGCTCTGG	TCAGGCTCAAGGCGACGGTAAG
	**Gb_18124**	GCCCGACTTGAGTGATGTTCCAG	CGCAGACCGTTGCAGAAGCTC
	**Gb_39589**	ATGTCTCCTTGGCTTCTGAGCCG	AACGCTCTCTCTCCTCGCCAA
	**Gb_29892**	TTCTGCTCCTATCCCGCTTCCC	ACGATAAGCCAACGCCCAATGAG
	**Actin (Arabidopsis)**	CTTAACCCAAAGGCCAACAGA	GCAAGGTCAAGACGGAGGAT
	**Actin (poplar)**	TTCTACAAGTGCTTTGATGGTGAGTTC	CTATTCGATACATAGAAGATCAGAATGTTC
	**Actin (ginkgo)**	CTGCCAAGGCTGTAGGTAAGG	TCAGATTCCTCCTTGATGGCG
