# Supplementary figures and images for: Dual function of GbNAC2 in flavonoid metabolism and hormonal pathways enhances salt tolerance in Ginkgo biloba
Source: For Res (Fayettev). 2025 Nov 20;5:e028. doi: 10.48130/forres-0025-0027 (PMC12648015; doi:10.48130/forres-0025-0027)

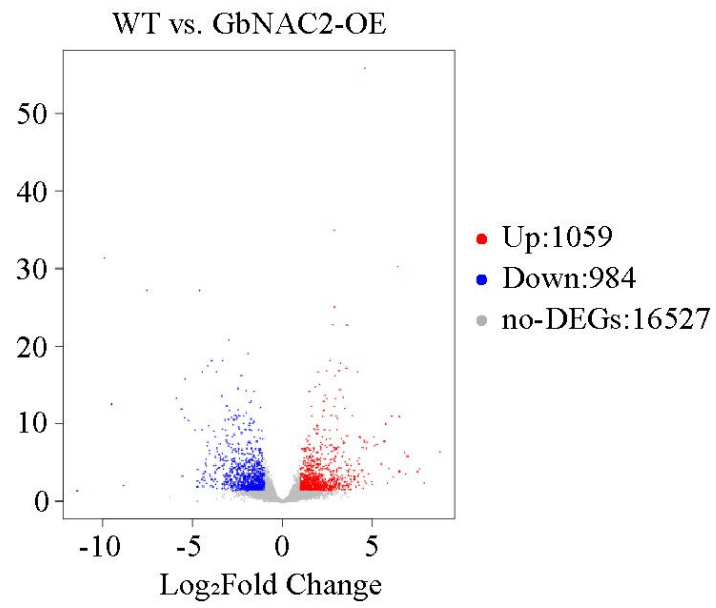

**Figure S3.** DEGs analysis in *GbNAC2* overexpression calli and WT calli.

Supplement: Supplementary file 1 — Supplementary data to this article can be found online. [file FR-2025-5-0027-Supplementary.zip › 10.48130_forres-0025-0027-Suppl-FigureS3.pdf]

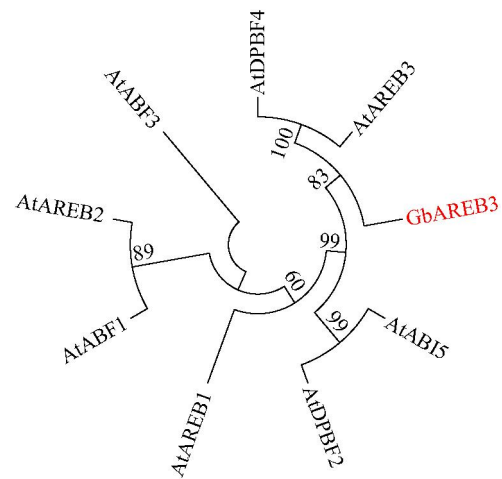

**Figure S5.** Phylogenetic relationships of *G. biloba* AREB3 and ABF family proteins in *Arabidopsis*.

Supplement: Supplementary file 1 — Supplementary data to this article can be found online. [file FR-2025-5-0027-Supplementary.zip › 10.48130_forres-0025-0027-Suppl-FigureS5.pdf]
